# Characterization of Anti-GAD65-Associated Neurological Syndromes: Clinical Features and Antibody Titers

**DOI:** 10.3390/neurosci5020015

**Published:** 2024-06-17

**Authors:** João Moura, Firmina Sambayeta, Ana Paula Sousa, Paula Carneiro, Esmeralda Neves, Raquel Samões, Ana Martins Silva, Ernestina Santos

**Affiliations:** 1 Department of Neurology, Hospital de Santo António, Centro Hospitalar Universitário do Porto, 4099-001 Porto, Portugalernestinasantos.neurologia@chporto.min-saude.pt (E.S.); 2 Department of Neurophysiology, Hospital de Santo António, Centro Hospitalar Universitário do Porto, 4099-001 Porto, Portugal; 3 Department of Immunology, Hospital de Santo António, Centro Hospitalar Universitário do Porto, 4099-001 Porto, Portugal; 4 Unit of Multidisciplinary Research in Biomedicine (UMIB), Instituto de Ciências Biomédicas Abel Salazar (ICBAS), University of Porto, 4099-001 Porto, Portugal; 5 Laboratory for Integrative and Translational Research in Population Health (ITR), 4099-001 Porto, Portugal

**Keywords:** anti-GAD65, limbic encephalitis, epilepsy, cerebellar ataxia, stiff-person syndrome

## Abstract

Introduction: Anti-GAD65 antibodies are associated with several neurological phenotypes. Antibody titers are increasingly recognized as useful in diagnosis and prognosis. Objective: To describe a Portuguese cohort of patients with anti-GAD65-associated neurological syndromes. Methods: Retrospective analysis of all patients with positive anti-GAD65 antibodies and associated neurological syndromes followed in a tertiary referral center. Results: Nineteen anti-GAD65 antibody-positive neurological patients were identified, 62.3% female, with a mean age of onset of 56.0 (SD = 13.3) years. Comorbid autoimmune disorders were present in seven patients. Six patients had limbic encephalitis (31.6%), four had epilepsy (21.1%), four had cerebellar ataxia (21.1%), and three had stiff-person syndrome (15.8%). Two patients presented with isolated cognitive dysfunction (executive and mnesic) in the absence of other neurological symptoms. The mean follow-up time was 24.0 (14.0–42.0) months, at the end of which the mean modified Rankin Scale (mRS) value was 2.0 (1.0–4.0). Screening for malignancies was negative in all patients. Serum quantitative analysis was carried out in 18 patients, 10 of whom showed titers above previously defined cut-off points (>10,000 IU/L for ELISA and >20 mmol/L for RIA). Quantitative CSF analysis was performed in nine patients, with four showing above-threshold titers. There was no association between anti-GAD65 levels and clinical phenotype or the final mRS values. High-dose intravenous methylprednisolone and oral prednisolone were the most common acute and chronic treatment regimens, respectively. Conclusion: Anti-GAD65 antibodies are associated with varied neurological syndromes, and antibody titers alone should not be used to exclude a disease.

## 1. Introduction

Glutamic acid decarboxylase (GAD) is a rate-limiting enzyme in the conversion of glutamate to gamma-aminobutyric acid (GABA) expressed in the central and peripheral nervous systems and in the pancreas [1,2]. Antibodies directed against the isoform 65 of GAD (anti-GAD65) have been correlated with several neurological syndromes [3]. Classically defined phenotypes include limbic encephalitis, cerebellar ataxia, epilepsy, and stiff-person syndrome [4,5,6]. However, there is still some concern about the possible implication of anti-GAD65 in atypical phenotypes, despite the majority of them being attributed to other neurological diseases discovered later by additional investigation [7].

The pathogenic role of anti-GAD65 is not clearly established since GAD65 is expressed intracellularly and thus inaccessible to circulating antibodies. It is still unknown whether anti-GAD65 directly causes antibody-mediated neuronal dysfunction [8] or if it is a marker of T-cell-mediated immunity [9], similar to autoantibodies mediating paraneoplastic neurological syndromes [10]. Recently, Muñoz-Lopetegi et al., (2020) showed that high serum anti-GAD65 titers (>10,000 IU/mL) correlated with more consensual phenotypes associated with the disease [11]. Moreover, these patients with a higher titer of anti-GAD 65 showed at least a partial response to immunotherapy, accompanied by a reduction in serum anti-GAD165. There is concern about attributing a neurological phenotype to low anti-GAD65 titers since these residual values are common in type 1 diabetes patients and healthy individuals [3]. However, even in low-titer circumstances, improvement after immunotherapy has been reported [12,13].

This study aims to characterize a Portuguese cohort of anti-GAD65-associated neurological syndromes.

## 2. Methods

### 2.1. Study Setting

This study included all patients followed in the neurology outpatient clinic of Centro Hospitalar Universitário do Porto (CHUPorto) with positive anti-GAD65 antibodies as registered by the Immunology Department’s database of the hospital. Data were retrospectively collected and analyzed from September 2020 to February 2022.

### 2.2. Clinical Data

Patient information was retrieved from the clinical records. The retrieved information included demographic and clinical data, such as date of birth, current age, gender, age of onset, age of diagnosis, clinical presentation, presence of other autoimmune disorders, immunosuppressive treatments used, follow-up time between the diagnosis and the time of data collection, and modified Rankin Scale (mRS) at the end of that period. Neurological syndromes were diagnosed by the respective practicing physician. Based on previous works, we defined classical anti-GAD65-associated neurological syndromes as limbic encephalitis, cerebellar ataxia, epilepsy, and stiff-person syndrome [4,5,6].

### 2.3. Anti-GAD65 Antibody Assays

Following the local protocol, anti-GAD65 was first tested as part of the antibody panel for autoimmune encephalitis using an indirect immunofluorescence assay of serum and CSF. Positive results were confirmed by immunoblotting. All patients with positive results in both tests were included. When available, paired serum and CSF samples were collected. Otherwise, serum samples drawn closest to the CSF tap were used. Some patients underwent further quantitative analyses in serum and CSF to determine the antibody titers using automated Enzyme-Linked Immunosorbent Assay (ELISA) and radioimmunoassay (RIA). The tests were conducted according to the manufacturer’s instructions and local protocols.

### 2.4. Statistical Analysis

For descriptive statistics, qualitative variables were studied using absolute and relative frequencies. For the quantitative variables, the mean and standard deviation, or median and interquartile range (p25–p75) (IQR), were calculated according to the normality of the distribution. A Mann–Whitney test compared the antibody titers and mRS between groups. A Chi-square was used to analyze the association between categorical variables.

IBM SPSS Statistics for Macintosh, version 27.0, was used for the analysis. A *p*-value inferior to 0.05 was considered statistically significant.

### 2.5. Ethical Approval

This study was authorized by the local Ethics Committee.

## 3. Results

In total, 19 anti-GAD65 antibody-positive patients with neurological symptoms were identified. Their characteristics are summarized in Table 1. There was a female predominance (62.3%), and the mean age of onset was 56.0 (SD = 13.3) years. Comorbid autoimmune disorders were present in seven patients; four patients presented with type 1 diabetes (21.1%), three with thyroiditis (15.8%), and two with myasthenia gravis (10.5%). Concerning neurological phenotypes, we identified six patients with limbic encephalitis (LE) (31.6%), four with epilepsy (21.1%), four with cerebellar ataxia (CA) (21.1%), and three with stiff-person syndrome (SPS) (15.8%). Additionally, three patients presented with diplopia, two with cognitive dysfunction, and one with parkinsonism. 

The mean age at onset by presenting phenotype in patients with classical syndromes ranged from 44.5 years in SPS to 54.7 years in LE patients. The median follow-up time was 24.0 (14.0–42.0) months, after which three patients died (21.4%): patient 1 died from cardiac arrest after a seizure; patient 4 died from respiratory infection; and patient 9 died from pulmonary embolism. The median modified Rankin Scale (mRS) value at the end of follow-up was 2.0 (1.0–4.0).

Two patients with cognitive dysfunction had this phenotype without any other neurological symptoms (patients 16 and 17). They were both females, presenting with executive dysfunction and memory impairment by ages 56 and 62. These symptoms were present for 5 and 2 years before the first consultation, respectively, and were confirmed by neuropsychological tests. EEG was normal in both, and brain MRI showed features of ischemic leukoencephalopathy in one patient and was normal in the other.

**Figure 1:**
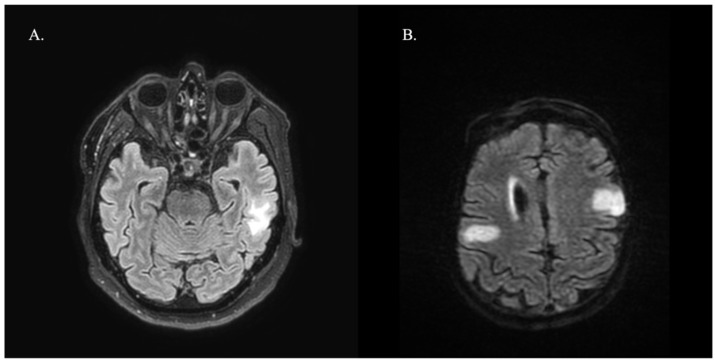
T2-weighted brain MRI with fluid-attenuated inversion recovery (FLAIR) hyperintense lesions attributable to autoimmune encephalitis related to anti-GAD65. (**A**) A patient with a temporal lesion involving the white matter that clinically presented with limbic encephalitis. (**B**) Parietal and frontal tumefactive lesions in a patient presenting with epilepsy.

Regarding neuroimaging, all patients had available brain MRI reports, showing T2/FLAIR hyperintensities in 47.4% of cases, most commonly in parietal (35.7%) and frontal (28.6%) locations. Patients diagnosed with LE showed mesial temporal sclerosis in two cases and hyperintense T2 lesions in the hippocampus and amygdala in three cases. Figure 1 shows two examples of brain MRI features. All patients were investigated with full-body CT and three patients with positron emission tomography (PET), which was negative for underlying malignancy. Besides anti-GAD65, patients tested negative for other antibodies in the indirect immunofluorescence panel. CSF pleocytosis was present in seven patients (36.8%) and >2 oligoclonal bands in six (31.6%).

Table 2 shows the descriptive analysis of patients presenting with classical phenotypes and their respective treatment regimens. A total of 18 patients (94.7%) had positive anti-GAD65 antibodies in serum and 15 (78.9%) had them in the cerebrospinal fluid (CSF). Serum quantitative analysis was carried out in 18 patients: 7 through ELISA and 6 through RIA. In total, 10 patients (52.6%) had anti-GAD65 titers above the previously defined high-level thresholds of >10,000 IU/L for ELISA [11] and >20 mmol/L for RIA [14]. Additionally, CSF quantitative analysis through ELISA was performed in nine patients (47.4%), obtaining a median value of 14.38 IU/L (2.9–7301.6). Four patients (21.1%) had a CSF anti-GAD65 antibody value >100 IU/L [11] (all with correspondingly above-threshold serum titers). Patients with both serum and CSF elevated titers of anti-GAD65 antibodies displayed LE and epilepsy as clinical phenotypes. From the patients presenting with cognitive dysfunction, one had elevated titers of anti-GAD65 antibodies in serum, and one had low titers but positive oligoclonal bands and pleocytosis in the CSF, supporting an immune-mediated process.

Regarding acute treatment modalities, eight (42.1%) patients received high-dose methylprednisolone (MPD) and seven (36.8%) patients received intravenous human immunoglobulin (IVIG). The most common maintenance modality was oral prednisolone (PD), which was used in seven (36.8%) patients, followed by rituximab in five patients (26.3%) and azathioprine in three patients (15.8%). Cyclophosphamide (CYC) was used in one case, and methotrexate (MTX) in another one. Nine patients (47.4%) required no chronic treatment to achieve disease control. We found no correlation between serum anti-GAD65 and the mRS values (*p* = 0.57) at the end of the follow-up period.

## 4. Discussion

The most common phenotypes associated with anti-GAD65 were LE, CA, and epilepsy, in line with the previously reported literature [11]. Only three cases of SPS were identified in this cohort, which might reflect that this entity is frequently misdiagnosed for other neurological conditions due to variable presentations [15].

Atypical or nonconsensual phenotypes are symptoms outside the spectrum of the previously defined group of established anti-GAD65-associated neurological syndromes (LE, epilepsy, CA, and SPS). In our sample, two patients diagnosed with cognitive dysfunction had positive anti-GAD65 antibodies. One could argue that these cases could, in fact, be LE. However, neurophysiological and imaging studies did not support this hypothesis. Furthermore, these patients complained of symptoms occurring for 2 to 5 years before the diagnosis, which is inconsistent with LE and more in line with dementia. Interestingly, a presentation consistent with subacute dementia has been previously reported in association with anti-GAD65 [16].

We also found patients presenting with parkinsonism and diplopia in combination with typical neurological syndromes. Parkinsonism has been previously described with CSF-positive anti-GAD antibodies [17]. Eye movement abnormalities have been previously described in association with anti-GAD65 antibodies [18,19], resulting in diplopia, as described in two patients from our series. The co-occurrence of other neurological involvement in anti-GAD65-associated neurological syndromes has been recognized in a considerable proportion of patients from previous cohorts [14]. Our study highlights the heterogeneity of these secondary neurological manifestations and the need for a better definition of these syndromes as a path to add to the understanding of anti-GAD65 pathophysiology.

A full-body CT yielded no underlying malignancy in any of the patients. This is in line with the knowledge that the anti-GAD65 antibody has a lower risk for a paraneoplastic cause [10]. The relatively high prevalence of other autoimmune disorders points to a systemic propensity for autoimmunity [20]. Despite this, demonstration of a direct pathogenic effect is still lacking. Approximately half of the patients had hyperintense T2 lesions in the brain MRI, a common finding in other anti-GAD65 cohorts [21]. The patients presenting with epilepsy had MRI lesions in topographies consistent with ictal semiology.

The literature clearly suggests a tendency towards higher titers in patients with classical syndromes, as shown in a cohort of 22 anti-GAD65 antibody-positive cases where patients with typical phenotypes had serum levels significantly higher than those with atypical presentations [7]. Our results point in the same direction, with 52.6% of our sample presenting values above the previously suggested cut-offs [11,14]. Two issues would arise with the rigorous application of this method to define anti-GAD65-mediated neurological syndromes: on the one hand, one patient with an atypical phenotype had a high anti-GAD65 titer; on the other hand, eight patients with low titers still had typical neurological syndromes. These findings warrant caution when guiding clinical decisions solely on the basis of anti-GAD65 levels in relation to a cut-off value. Furthermore, these patients with classical anti-GAD65-associated neurological syndromes with low antibody titers still showed a positive response to immunosuppressive therapy in previously reported series [12]. This notion is particularly relevant, considering that anti-GAD65 antibodies are not established as directly pathogenic. We found no association between anti-GAD65 initial levels and disability (measured through the mRS) at the end of the follow-up period. Two previously reported studies also found no association with disease severity or response to immunosuppressive therapy (IVIG and rituximab) [22,23]. However, these studies used quality-of-life measurements as an outcome, which differs from the mRS used in this cohort. In contrast with these findings, a more recent study showed an association between anti-GAD65 levels and a response to immunosuppressive therapy accompanied by a reduction in anti-GAD65 titers, suggesting its use in monitoring treatment response [11]. In the future, it would be interesting to measure anti-GAD65 titers as a monitoring strategy at follow-up.

MPD was the mainstay of acute treatment, followed by IVIG. In the literature, there is a low level of evidence regarding the use of MPD for anti-GAD65-associated neurological syndromes [24]. In most cases in which it was used, patients presented with features of an immune-mediated SNC disorder with no knowledge about the associated antibody ad initium. As such, a cycle of MPD was initiated, and later, the therapeutic regimen was tailored. Regarding maintenance immunosuppressive therapy, our cohort replicates a previous case series [11]. Clinical stabilization with immunosuppressive treatment appears to be the rule [25].

This study has several limitations that should be considered. Its retrospective design with a relatively small sample size prevents us from establishing casual associations between phenotype, antibody titers, and outcome. The quantitative analysis of anti-GAD65 was conducted by two different methods; thus, we are unable to uniformize the analysis. Furthermore, CSF analysis for anti-GAD65 quantification, which usually provides the strongest proof for the neurological relevance of these antibodies, was only performed in approximately half of cases [11]. No consistent measurements of disability (mRS) were conducted throughout the disease course and therapeutic interventions, and so we were only able to collect data on the mRS at the end of the follow-up period.

In the future, it would be interesting to study anti-GAD65-associated neurological syndromes prospectively, with particular attention to atypical disease manifestations.

## 5. Conclusions

Our study characterizes a Portuguese cohort of anti-GAD65-associated neurological syndromes. Most cases present classical phenotypes (LE, CA, epilepsy, and SPS), but we highlight the occurrence of atypical manifestations, such as isolated cognitive dysfunction. Although patients with classical phenotypes typically show high antibody titers, the possibility of anti-GAD65-associated disease should not be excluded based on this finding alone.

## Figures and Tables

**Table 1:** Characterization of the patients with anti-GAD65-associated neurological syndromes.

Case	Age of Onset	Sex	Clinical Presentation	Clinical Presentation	Anti-GAD65 Serum (U/mL) and Method	Anti-GAD65 CSF (U/mL) *	FU	mRS
1	63	M	LE	Subacute altered mental status, personality changes, and working memory impairment	22,178.70 ELISA	-	12	6
2	52	F	CA	Gait unsteadiness, falls	104,234.40 ELISA	-	36	4
3	49	F	CA	Gait unsteadiness, dysmetria	2.26 RIA	2.61 ELISA	16	4
4	74	M	CA, P	Gait unsteadiness, parkinsonism	41.79 RIA	3.13 ELISA	16	6
5	24	F	LE	Refractory status epileptic, altered mental status, dystonic posture of the left arm	2000.00 ELISA	-	18	1
6	79	M	LE	Subacute working memory impairment and frontal syndrome	35,419.20 RIA	2610.20 ELISA	12	2
7	65	M	LE	This patient had a history of perinatal strokes. By age 55, he had started developing seizures and cognitive dysfunction	14.10 RIA	-	108	4
8	58	F	SpS, D	Stiffness, muscle spasms, diplopia	274.64 RIA	-	132	3
9	75	F	LE	Personality changes and seizures, subacute onset	123.68 RIA	-	24	6
10	69	F	Ep	Generalized seizures	110,530.30 ELISA	19,964.00 ELISA	48	1
11	45	F	SpS, D	Stiffness, muscle spasms, diplopia	0.50 RIA	10.64 ELISA	24	2
12	56	M	Ep	Generalized seizures	0.50 RIA	0.57 ELISA	32	2
13	49	M	CA, D	Gait unsteadiness, dysmetria, diplopia	2.34 RIA	-	96	4
14	52	F	SpS	Stiffness, muscle spasms	-	-	32	2
15	52	F	LE	Subacute altered mental status, personality changes, and working memory impairment	101,572.00 ELISA	11,993.00 ELISA	12	1
16	62	F	Dementia	Altered mental status and working memory impairment	174,740.10 ELISA	-	11	1
17	56	F	Dementia	Personality changes and working memory impairment	155.70 RIA	-	4	2
18	59	F	Ep	Focal motor seizures	209,461.70 ELISA	1735.91 ELISA	24	1
19	39	M	Ep	Generalized seizures	0.50 RIA	14.38 ELISA	60	2

F—female; M—male; LE—limbic encephalitis; Ep—epilepsy; CA—cerebellar ataxia; SpS—stiff-person syndrome; D—diplopia; P—parkinsonism; CSF—cerebrospinal fluid; FU—follow-up; mRS—modified Rankin Scale. *—antibody titers in CSF were determined by Enzyme-Linked Immunosorbent Assay (ELISA).

**Table 2:** Characterization of patients with classical anti-GAD65-associated neurological syndromes.

Characteristics	Sample
Demographic	
Female, number (%)	12 (63.2)
Age of onset, mean (SD)	56.0 (13.3)
Age of diagnosis, mean (SD)	54.4 (14.6)
Clinical phenotype	
Ep, number (%)	6 (31.6)
LE, number (%)	6 (31.6)
CA, number (%)	4 (21.1)
SpS, number (%)	3 (15.8)
Serostatus	
Anti-GAD65 positive, serum, number (%)	17 (89.5)
Anti-GAD65 positive, CSF, number (%)	15 (78.9)
Therapeutic regimen	
MPD, number (%)	8 (42.1)
IVIG, number (%)	7 (36.8)
RTX, number (%)	5 (26.3)
AZA, number (%)	3 (15.8)
MTX, number (%)	1 (5.3)
CYC, number (%)	1 (5.3)
Maintenance PD, number (%)	7 (36.8)
Follow-up time (months), median (IQR)	24.0 (14.0–42.0)
Final mRS, median (IQR)	1.0 (1.0–4.0)

IQR—interquartile range; LE—limbic encephalitis; Ep—epilepsy; CA—cerebellar ataxia; SpS—stiff-person syndrome; CSF—cerebrospinal fluid; mRS—modified Rankin Scale; MPD—methylprednisolone; PD—prednisolone; IVIG—intravenous immunoglobulin; AZA—azathioprine; MTX—methotrexate; CYC—cyclophosphamide.

## Data Availability

The generated data can be made available upon reasonable request to the authors.
